# Complete Genome Sequence of the *Streptomyces* Phage Nanodon

**DOI:** 10.1128/genomeA.01019-16

**Published:** 2016-10-06

**Authors:** Ivan Erill, Steven M. Caruso

**Affiliations:** Department of Biological Sciences, University of Maryland, Baltimore County, Baltimore, Maryland, USA

## Abstract

*Streptomyces* phage Nanodon is a temperate double-stranded DNA *Siphoviridae* belonging to cluster BD1. It was isolated from soil collected in Kilauea, HI, using *Streptomyces griseus* subsp. *griseus* as a host.

## GENOME ANNOUNCEMENT

*Streptomyces* spp. are Gram-positive, filamentous, and spore-forming members of the *Actinomycetaceae* family, from which two-thirds of antibiotics have been derived (1). Here, we present the genome of the double-stranded DNA bacteriophage Nanodon, a *Siphoviridae* isolated from volcanic soil collected in Kilauea, HI (22°10′28.6″N 159°26′08.3″W), which was then harvested and investigated using *Streptomyces griseus* subsp. *griseus* (ATCC 10137) (*S. griseus*) as a host by University of Maryland, Baltimore County (UMBC) undergraduate researchers, as described previously (2).

Nanodon produced variably sized clear plaques when incubated for 48 h on lawns of *S. griseus* grown on supplemented nutrient agar at 30°C (3). Nanodon has an icosahedral capsid (d = 46 nm) with a flexible noncontractile tail (l = 151 nm). Shotgun sequencing was carried out by the Pittsburgh Bacteriophage Institute to approximately 1,201-fold coverage by Illumina sequencing. Nanodon has a linear 50,082-bp genome with an 11-bp 3′ sticky overhang and a 65.7% G+C content. Genomic analysis of Nanodon determined that the phage contains 75 protein-coding genes, of which 25 have been assigned predicted functions. Among these were both a serine integrase and an immunity repressor, suggesting that Nanodon is a temperate phage (4, 5).

Genomic comparison of Nanodon with the 69 *Streptomyces* phages sequenced to date by SEA-PHAGES participants (2) identified Nanodon as a member of cluster BD, one of nine established *Streptomyces* phage clusters (http://phagesdb.org/) and, specifically, as a member of subcluster BD1. Nanodon shares 84.4% ± 0.02% average ± standard deviation (SD) nucleotide identity (ANI) with other members of subcluster BD1 available in GenBank, and 71.5% ± 0.02% ANI with phages in the other four current BD subclusters (6). A comparison of Nanodon by whole-genome alignment (7) and phylogenetic analysis using the terminase gene also demonstrated that Nanodon clusters with other members of the BD1 subcluster.

Codon usage bias (CUB) analysis using the scnRCA program (8) and reference sets derived from several *Firmicutes* and *Actinobacteria* genomes revealed that protein-coding sequences in the Nanodon genome are adapted for translational optimization on *Streptomyces* hosts, with capsid and tail assembly proteins showing the highest level of codon optimization. However, CUB patterns revealed no specific optimization for *S. griseus* compared to other *Streptomyces* species, suggesting that Nanodon might have broad-range specificity within the *Streptomyces*, as seen previously for other temperate *Streptomyces* phages (9, 10).

### Accession number(s).

The complete genome sequence of the *Streptomyces* phage Nanodon is available in GenBank with the accession no. KX344445.
